# Research skills in medical education

**DOI:** 10.15694/mep.2018.0000151.1

**Published:** 2018-07-23

**Authors:** Helen R Watson, Steven Burr

**Affiliations:** 1 Peninsula Medical School

**Keywords:** research skills, transferable skills, life-long learning, employability

## Abstract

This article was migrated. The article was marked as recommended.

The ability to find, interpret and use information is a key skill in any clinician’s arsenal. During medical training, we aim to equip our students with the ability to navigate the potentially baffling amount of information available online, and come to valid conclusions. This article reflects on the nature of research skills and how they are delivered in medical education. It also explores whether these are the most efficient methods for enabling students to become able researchers. Comparisons with other types of university degrees are made, and consideration given to how research skills should best be integrated into our teaching.

## How can we best prepare our students to enquire, evaluate and be life-long learners?

Research skills are required in all branches of medicine ( Laidlaw *et al.*, 2012). Clinicians need to be lifelong learners, able to evaluate evidence and understand the process of scientific enquiry. Whether or not a doctor pursues a research career, they still need to be able to make sense, and be critical, of the huge amount of information available online. They may also be required to carry out their own research study, and the training we provide should lay the foundations for them to develop their own rigorous and informed approaches to research.

Knowledge, skills and behaviours can be absorbed by students without them noticing, and it is only when the values they are exposed to conflict with their own that they become conscious they have been taught covertly ( Phillips, 2013). This approach to conveying information may be more appropriate for some transferable skills than others, and carries the danger that values can be imparted on students without them considering if they fully agree with or understand them. Research skills include tangible principles that can be taught more visibly; like good study design and the scientific method. Evidence suggests that we are more likely to recognise having learned something if we are told explicitly that we will be learning it ( Murdoch-Eaton *et al.*, 2010). In addition, enabling students to be more aware of their own skillsets may well be important for employability, as they will be able to communicate these skillsets to potential employers when applying for jobs. In a typical academic setting, teachers may have spent decades with research as their primary activity. In contrast, the students’ primary activity is to learn and understand medical knowledge. Thus, we may need to be more overt about research skills and explain their impact on employment within our curricula so that the students can understand what they are learning, and why.

As educators, we must understand the perceptions of research that students have when they enrol in medical school. Most of our medical students are high-achievers, previously attaining high grades by learning and recalling facts. A UK study found that whilst school pupils are generally familiar with the idea of research, for example in forming hypotheses, they have few opportunities to test their own research questions ( Yeoman *et al.*, 2017). Whilst considering this evidence, it is also important to note that many of our students in the UK are now graduates, coming from a first degree where they are likely to have been exposed to research experience. We must consider what, if any, exposure to research our students have had before coming to medical school, especially when we ask students to conduct novel research or literature reviews in the early years of medical study. We should also be aware that science education prior to university is predominantly factual, leading to a potential distortion of the amount of uncertainty there is in science. It is important to openly explore the idea that ‘nobody knows’ as a new and uncomfortable concept for those unfamiliar with research. Therefore, it might not be appropriate to ask students to interpret peer-reviewed primary research until they understand the process of research and the limitations of knowledge. To ease this transition, we should emphasise application, and create employment-relevant contexts to engage students in developing these skills ( Murdoch-Eaton and Whittle, 2012).

In undergraduate science courses, research is generally introduced in a constructive fashion, culminating in a final year extended project within a research framework ( Willison and O’Regan, 2007). Factual knowledge is taught in the early years of scientific study, alongside non-explorative, tried and tested experiments. Opportunity for novel research and exploration of the literature comes in the final year. In contrast, during the early years of medical study we often expect students to evaluate research (e.g. with problem-based learning). Perhaps we should learn from traditional science courses and introduce research skills teaching in a more constructive way, building knowledge and understanding each year. However, an extensive US study argues that although science students are exposed to research opportunities, they are predominantly assessed on factual content at lower cognitive levels, meaning that graduates are less equipped with transferable skills than they perhaps should be ( Momsen *et al.*, 2010). Aligning assessment with transferable skills is key, but it is also difficult, due to the less tangible nature of transferable skills ( Laidlaw *et al.*, 2012). Science courses may need to be more explicit about research skills in the curriculum, and perhaps medical students need a more constructive introduction to research. What is clear is that both science and medical curricula must strive for more effective assessment of research skills.

In order to become lifelong learners, medical students must understand scientific concepts, but also have the skills to find, filter and use new information. With near instant access to extensive knowledge using modern technology, is it still appropriate to ask our students to recite normal range values, drug names and doses? Furthermore, in 40 or so years of practice, guidelines, drugs, surgical procedures and many other things will change. For example, all clinicians throughout their careers require critical thinking and the ability to deal with uncertainty, whereas the same is not true for some aspects of physiology and pharmacology. Of course, this is not to say we should not teach the sciences, but that the priority should shift from learning facts to developing the skills required to evaluate knowledge. This is not a proposal to teach research skills in a standalone way, rather to continue to teach them in context with social interaction ( Berkhout *et al.*, 2018), whilst moving the emphasis away from factual recall.

Most university courses claim to be research led, teaching the most recent discoveries in their fields, and we should extend this to research skills training. Styles of oral communication between people may change gradually over centuries, thus it is likely that a doctor can use the same communication strategies throughout their career. In contrast, the skills required to conduct and access research are constantly changing, due to huge advances in technology and internet access and because of the vast increase in research output now available. Over the past 15 years, the methods we use to access scientific journals have changed immensely. It is clear that a lack of training is a barrier to clinicians finding the information they need ( Davies, 2007), whilst they must also possess the critical ability to not believe everything they read online ( Bullock, 2014). Therefore to future-proof our doctors, we must help them develop the ability to utilise technology effectively to interrogate massive databases, independently of format.

## Take Home Messages

When considering research skills in the curriculum, we must be proactive in responding to the different educational backgrounds of our students, and changes in demands on clinicians. We need to equip students with the agility to become lifelong learners, aware of their own skillsets and able to adapt to change.

## Notes On Contributors

Dr Helen R Watson is a Lecturer at Peninsula Medical School, University of Plymouth.

Dr Steven A Burr is an Associate Professor and Deputy Director of Assessment for Medicine and Dentistry at Peninsula Medical School, University of Plymouth.
