# Supplementary material for: Research skills in medical education
Source: MedEdPublish (2016). 2018 Jul 23;7:151. [Version 1] doi: 10.15694/mep.2018.0000151.1 (PMC10701811; doi:10.15694/mep.2018.0000151.1)
Supplement: Peer review history

# Reviewer report: Reviewer response for version 1

**DOI:** 10.21956/mep.19616.r29166

**Published:** 2018-08-09

**Authors:** Irine Sakhelashvili

**Affiliations:** 1 European University

This review has been migrated. The reviewer awarded 4 stars out of 5 I was reading this brief, but concise article touching, very thought-provoking topic with great interest. As my previous colleagues mentioned, yes, perhaps research skills are required only for researchers. However, by my opinion, it may be concerning to the advanced abilities and knowledge, like MatLab, Spss, etc. In the matter of the critical thinking, reading and writing skills, (and not only them) they are absolutely necessary in every field of medicine, regardless, are you theoretic or doctor-practitioner. I strongly believe that the ability of the logical reasoning, ability to find the relevant literature, to distinguish valid and invalid arguments is useful for everyone, especially for the doctors. Moreover, familiarity with the common guidelines and ethical principles, which are the pillars in the scientific society must be valuable for all the students of medical faculty. Maybe basics of research are not easy for beginners, but Anatomy and physiology also is not easy for studying...I share the opinion of the respectful authors that we should teach the students each year giving them the opportunity to come deeper into the research area. Fundamental principles of the research process and basic language of scientific society must be an obligatory part of the curricula. Not only scientist should read and analyze as much as possible. Professionals, especially doctors should be in touch with the state-of-the art technology, with novel treatment strategies, with new approaches in a field of their occupation. Evidence-based medicine practice needs to be familiar with the strategies of seeking the information and filter relevant evidence from the big ocean of data. In respect of this, I agree with the authors that instead of teaching only the theory, it would be better to help them in obtaining the critical appraisal skills. Developing the abilities of independent work would be much helpful for the future career of the medical students.

Reviewer Expertise:

NA

No decision status is available

# Reviewer report: Reviewer response for version 1

**DOI:** 10.21956/mep.19616.r29167

**Published:** 2018-08-03

**Authors:** Sateesh Babu Arja

**Affiliations:** 1 Avalon University School of Medicine

This review has been migrated. The reviewer awarded 4 stars out of 5 I agree with Professor Subha Ramani’s comments. All medical students do not require research skills unless they choose the research career. But everyone should know Basic research principles, research methodology, and research ethics. They should be able to read, review, appraise and critique the literature to improve the practice of Medicine. This is all about practice-based learning which is one of the core learning objectives of medical education. Students should have lifelong learning aptitude. One thing I would like to see in this article is if there are any solutions. Journal club sessions in early medical education where medical students practice appraising and critiquing the literature, Evidence-based medicine courses in the early years of medical school, and problem-based learning strategies can be solutions.

Reviewer Expertise:

NA

No decision status is available

# Reviewer report: Reviewer response for version 1

**DOI:** 10.21956/mep.19616.r29164

**Published:** 2018-07-25

**Authors:** Subha Ramani

**Affiliations:** 1 Harvard Medical School, Brigham and Women's Hospital

This review has been migrated. The reviewer awarded 3 stars out of 5 I agree with many of the points made by the authors. If physicians in training are required to be able to sit through reams of evidence, critically appraise and know what and when to apply in their own practice, they need to be trained in the skills reported in this article. We live in an era of ever increasing information overload, unfortunately not all the information is scientific or accurate. Thus, all physicians need to sort through the wheat from the chaff and know what data are applicable to their own clinical practice. In addition, patients retrieve information from the internet and come into consultations with this information. Physicians need to be prepared to engage in these conversations and inform patients what information is valid and what is not. I agree that physician trainees need these skills regardless of whether they pursue a research career or not because they need critical appraisal skills to practice high quality patient care and engage in academic discussions with their peers. I wonder, though, if everyone needs research skills or the skills to appraise research and practice evidence based patient care. In-depth 'research' skills may be necessary for those planning research careers. Those who do not intend to be researchers need the skills of retrieval, appraisal and application definitely. Lastly, I agree that knowledge tests will not assess adequately understanding or application.While I agree with the principles, I would like to see more solutions to these challenges and hope the authors or others would educate other educators on how to effectively and efficiently achieve these goals.

Reviewer Expertise:

NA

No decision status is available

# Reviewer report: Reviewer response for version 1

**DOI:** 10.21956/mep.19616.r29165

**Published:** 2018-07-23

**Authors:** P Ravi Shankar

**Affiliations:** 1 American International Medical University

This review has been migrated. The reviewer awarded 4 stars out of 5 This is an interesting brief article dealing with the teaching-learning of research skills in medical education. Medical students and doctors should have the skill to evaluate the ever increasing amounts of information easily available through modern technology. The amount of information is likely to accelerate in the future. In a situation where information is readily accessible and can be looked up easily it may not make sense to have students memorize information which can be easily retrieved. I agree with the authors that the emphasis should be on concepts and less on facts. Modern research highlights the fact that there is uncertainty in medicine and there are areas where knowledge is deficient. Many medical schools teach research skills to their students but I am not sure that students gain knowledge of the research process and the limitations of knowledge. Inculcating lifelong learning skills in students is important so that they are ready to face the challenges of the ever changing medical landscape of tomorrow. I agree with the authors that the emphasis should shift from learning facts to learning to evaluate knowledge.

Reviewer Expertise:

NA

No decision status is available
